# Clinical heterogeneity of abnormal glucose homeostasis associated with the *HNF4A* R311H mutation

**DOI:** 10.1186/1824-7288-40-58

**Published:** 2014-06-19

**Authors:** Maurizio Delvecchio, Rosa Di Paola, Davide Mangiacotti, Michele Sacco, Claudia Menzaghi, Vincenzo Trischitta

**Affiliations:** 1 Dipartimento di Scienze e Chirurgia Pediatriche, U.O. “B Trambusti”, A.O.U. Consorziale Policlinico Giovanni XXIII, Bari 70100, Italy; 2 Research Unit of Diabetes and Endocrine Diseases, IRCCS Casa Sollievo della Sofferenza, San Giovanni Rotondo (FG) 71013, Italy; 3 Paediatrics Unit IRCCS Casa Sollievo della Sofferenza, San Giovanni Rotondo (FG) 71013, Italy; 4 Department of Experimental Medicine, ‘Sapienza’ University, Rome 00161, Italy

**Keywords:** Diabetes mellitus, *HNF4A*-MODY, Monogenic diabetes, Children, Adults, Gestational diabetes, Type 2 diabetes

## Correspondence

Dear Sir,

Maturity Onset Diabetes of the Young (MODY; MIM#606391) represents a genetically and clinically heterogeneous form of diabetes mellitus (DM) [1-3], characterized by hyperglycaemia or overt diabetes, in at least two or three consecutive generations, onset <25 years of age, absence of anti ß-cells antibodies. Loss-of-function *HNF4A* mutations cause a progressive loss of ß-cell function [4] and, eventually, frank hyperglycaemia denominated as MODY1. *HNF4A* mutations have been associated with MODY1, type 2 diabetes and gestational diabetes (GDM) [5].

### Findings

We describe a 10 years old diabetic girl recruited within a sample of MODY patients [6], Personal history: she is the first of two children born from non-consanguineous parents and was born full term and appropriate for gestational age after a pregnancy complicated by GDM. No transient congenital hyperinsulinism was detected [7]. She was healthy until the age of 9.1 years, when she was admitted to the local Hospital because of fainting. At admission, the following was recorded: blood glucose of 200 mg/dl; blood pressure, fundus oculi, heart ultrasound, electrocardiogram, blood biochemistry and pH were all normal; anti-thyroid and anti ß-cells antibodies were negative; finally, oral glucose tolerance test (OGTT) was consistent with DM (Table 1), with reduced insulin production (Matsuda Index 17.18, HOMA-IR 0.98, Insulinogenic Index −0.02, Disposition Index 0). Familial history was as follows: 40 years old healthy father; 35 years old mother, diagnosed as having GDM in both pregnancies (during pregnancies fasting blood glucose ranging from 110 to 140 mg/dl, blood glucose at 120′ of OGTT 597 mg/dl and 403 during the first and the second pregnancy, respectively); a 6 years old healthy brother; maternal grandmother and one out of 2 maternal uncles with type 2 DM which was diagnosed when he was 30 years old.

**Table 1:** The table shows clinical and biochemistry data of the proband at DM diagnosis in the upper part and data at last examination of all mutation carriers (including proband) in the lower part

**Proband’s data at the time of clinical diagnosis of DM (i.e. at age of 9.1 years)**				
**HbA1c (mmol/mol)**		58		
**C-peptide (ng/ml)**		2.9		
**Total/HDL cholesterol (mg/dl)**		176 / 34		
**Triglycerides (mg/dl)**		105		
**Systolic/diastolic blood pressure (mmHg)**		113 / 65		
**Fundus oculi**		Normal		
**Anti-thyroid, ICA, GAD, IA-2 antigens antibodies**		Negative		
**Oral glucose tolerance test**		**Blood glucose (mg/dl)**	**Blood insulin (microU/ml)**	
**Before glucose load**		100	10.4	
**30 min after glucose load**		215	57.2	
**60 min after glucose load**		308	82.2	
**90 min after glucose load**		351	84.3	
**120 min after glucose load**		320	77.7	
**Clinical data**	**Proband**	**Brother**	**Mother**	**Uncle**
**Age at examination (yrs)**	10.7	8	36.5	33.1
**Age at diagnosis (yrs)**	9.1	7.2	26	30.2
**Glycaemic status diagnosed**	Transient DM	IGT	GDM	DM
**Age at genetic testing (yrs)**	9.6	7.2	35.9	32.5
**BMI (Kg/m**^**2**^**)**	19	19.5	20.9	26.3
**Blood pressure (mmHg)**	107 / 68	110 / 60	160 / 90	130 / 80
**HbA1c (mmol/mol)**	6.1	37	40	60
**Fasting blood glucose (mg/dl)**	99	72	80	171
**C-peptide (ng/ml)**	1.9	1.3	1.1	1.4
**Insulin (microU/ml)**	7.6	3.5	3	4.3
**Total cholesterol (mg/dl)**	157	126	152	177
**HDL cholesterol (mg/dl)**	57	35	55	50
**LDL cholesterol (mg/dl)**	80	77	83.8	118
**Triglycerides (mg/dl)**	59	54	66	45
**Creatinine (mg/dl)**	0.45	0.59	0.67	0.89
**Albuminuria (mg/dl)**	7	8.4	3	8.2
**Glycosuria (mg/dl)**	Absent	Absent	Absent	Absent
**Anti-hyperglycaemic therapy**	None	None	Diet, physical activity	Diet, physical activity
**Fundus oculi**	Normal	Normal	Normal	Normal

DM: diabetes mellitus. IGT: impaired glucose tolerance at OGTT. GDM: gestational diabetes mellitus.

At admission, two IU of regular insulin at lunch and at dinner and a hypocaloric diet (BMI 24.1 kg/m^2^, 95th centile) were started. Three weeks later, body weight had dropped 1 kg and insulin injections were stopped because of recurrent hypoglycaemias. The patient was referred to our Unit 5 months later, still off of insulin. At that time, HbA1c was 40 mmol/mol and BMI 19.4 kg/m2 (75th centile). The HNF4A (NM_175914.4) gene analysis by PCR followed by direct sequencing showed the p.R311H c.932G > A variation in exon 8 (previously reported as p.R323H [8]). This mutation resides in the highly conserved extreme carboxy terminal domain [9] and causes a semiconservative aminoacid substitution predicted to be probably damaging by The Human Gene Mutation Database (http://www.hgmd.org) [10] and by PolyPhen-2 tool [11]. Genetic analysis was carried out also in first-degree relatives and in the maternal uncles, showing the same mutation in the mother, the brother, and the diabetic uncle. They underwent a comprehensive laboratory evaluation (Table 1). When 7.9 year-old, the brother underwent further examination showing impaired glucose tolerance (IGT) (baseline and after 120′ blood glucose and insulin: 78 and 151 mg/dl, 6.4 and 58.7 ng/ml, respectively), low insulin production (Matsuda Index 28.56, HOMA-IR 0.77, Insulinogenic Index −0.05, Disposition Index 0), HbA1c 39 mmol/mol, and BMI 20.4 kg/m^2^ (90th centile).

## Conclusions

*HNF4A* mutations lead to a progressive decrease of insulin secretion and hyperglycaemia [12] requiring oral hypoglycaemic drugs or insulin in most cases [13]. Our proband required low insulin dose (0.09 IU/kg/day) only for few weeks. This transient course is puzzling and never described in *HNF4A-*MODY children so far. Likely, in the weeks before the diagnosis, some triggering factors played a role in deteriorating glucose homeostasis. As she became normoglycaemic and insulin free after some weight lost, we suggest that overweight may have played a major role, but we cannot exclude the role of other concomitant factors (stress or infectious diseases). Currently the proband, 11-year-old, is on normocaloric diet and presents a good glycaemic control (HbA1c 40 mmol/mol) without any hypoglycaemic treatment.

We did not perform any molecular study showing the effect of the mutation, so no direct effect can be postulated. However, we show that it segregates with hyperglycaemia, as it was found in the mother (GDM, currently normoglycaemic and normal weight), in the maternal uncle (type 2 DM onset at 30 years of age, currently overweight, diabetic and on hypoglycaemic diet), and in the brother (IGT). The same mutation was previously described in a 46 year-old obese man with type 2 DM and nephropathy [8]. Later, in the same position, a mutation with Arginine replaced by Cysteine (previously reported as p.R324C), was described in a 13 year-old Japanese MODY patient [14]. Unfortunately, no information about the families were reported in both cases, making impossible to speculate about any role of *HNF4A*-R311H on glucose homeostasis. To better assess its pathogenic role, we screened 198 non-diabetic individuals and 138 type 2 DM patients without family history of autosomal dominant inheritance of hyperglycaemia / diabetes and negative data were obtained.

The *in silico* analysis suggests that the R311H mutation causes a semiconservative aminoacid substitution predicted to be probably damaging [10,11]. Since some studies indicates a neutral [15], and others a deleterious [16] effect of such variation on HNF4A transcriptional activity, its biological role is controversial.

In conclusion, we report on a family with the *HNF4A*-R311H mutation cosegregating with heterogeneous phenotype of abnormal glucose homeostasis, including MODY1. The lack of a clear genotype-phenotype association requires great caution before considering this mutation causative of MODY1. We suggest that also transient DM warrants the screening for MODY in the presence of indicative family history, even if with clinical heterogeneity of abnormal glucose homeostasis.

## Consent

Informed consent was obtained from the patient's parents for publication of this case report.

## Abbreviations

DM: Diabetes mellitus; IGT: Impaired glucose tolerance; OGTT: Oral glucose tolerance test; GDM: Gestational diabetes mellitus.

## Competing interests

The authors declare that they have no competing interests.

## Authors’ contributions

DM and RDP carried out the molecular genetic studies and participated in the sequence alignment. MD and VT participated in the clinical care and investigations of children and adults. MD and CM conceived the case report. VT, RDP, and MS coordinated and helped to draft the manuscript. MS and CM drafted the manuscript. All authors read and approved the final manuscript.
